# Availability of Bedside and Laboratory Testing for Carbon Monoxide Poisoning in the Upper Midwestern United States

**DOI:** 10.5811/westjem.2019.2.41428

**Published:** 2019-04-16

**Authors:** Thomas Masters, Brian Willenbring, Bjorn Westgard, Jon Cole, Stephen Hendriksen, Joseph Walter, Christopher Logue, Travis Olives

**Affiliations:** Hennepin Healthcare, Department of Emergency Medicine, Minneapolis, Minnesota

## Abstract

**Introduction:**

The objective of this study was to assess the ability to test patients for carbon monoxide (CO) exposure in all hospitals in three United States (U.S.) Midwestern states.

**Methods:**

We surveyed hospitals in three states. Telephone queries assessed processes for measuring carboxyhemoglobin, including capacity for real-time vs send-out testing. Facilities were separated based on their location’s population size for further analysis. Descriptive statistics are reported.

**Results:**

Of the 250 hospitals queried, we ultimately excluded 25. Nearly all (220, 97.8%) reported a process in place to test for CO exposure. Over 40% (n=92) lacked real-time testing. Testing ability was positively associated with increasing population size quartile (range 32.6% – 100%). Hospitals in the lowest-quartile population centers were more likely to report that they were unable to test in real time than those in the largest-quartile population centers (67.4% vs 0%).

**Conclusion:**

In a large geographic region encompassing three states, hospital-based and real-time capacity to test for CO exposure is not universal. Hospitals in smaller population areas are more likely to lack real-time testing or any testing at all. This may have significant public health, triage, and referral implications for patients.

## INTRODUCTION

Carbon monoxide (CO) is an odorless and invisible gas that may not be apparent to individuals exposed to it. Yet as a byproduct of combustion from sources such as furnaces, heaters, and engines, CO is pervasive in modern life. CO poisoning occurs when an individual is exposed to the gas at sufficient concentrations to cause symptoms with or without end-organ dysfunction. It is one of the leading causes of poisoning in the United States (U.S.) and around the world.^1^ It has been estimated that CO poisoning is responsible for 50,000 emergency department (ED) visits in the U.S. annually.^2^ Public health and legislative efforts have sought to increase awareness of CO poisoning and the use of CO detectors. This has contributed to fewer ED visits and deaths, particularly among intentional exposures. However, accidental exposures have diminished at a slower pace, and the rate of hospitalizations for CO poisoning remains essentially unchanged.^3^–^6^

Legislation requiring CO detectors in certain settings has helped to make significant environmental exposures less frequent and, when present, more apparent to clinicians.^7^,^8^ In the absence of scene alarms or source exposure history, the vague and nonspecific nature of presenting symptoms can make diagnosis a challenge. Patients may present with symptoms ranging from headache and dizziness, nausea and vomiting, to coma.^9^ While history and physical findings may point to the diagnosis, clinicians must maintain a high degree of suspicion. A missed diagnosis can have significant consequences, as CO poisoning can cause acute and persistent neurologic and cardiac injury,^10^ and therapy, whether with normobaric or hyperbaric oxygen, must be initiated in a timely manner.^11^ It is recommended that the diagnosis of CO poisoning should be confirmed by detecting an elevated carboxyhemoglobin (HbCO) level in the context of clinical symptoms.^12^,^13^ In the absence of real-time testing, therapy, hospitalization and referrals may be necessary based on clinical suspicion alone.

In the U.S., two common methods to detect HbCO in poisoned patients are a venous blood assay and finger CO-oximetry. A blood assay is the oldest method, but requires a laboratory equipped to perform the test.^14^,^15^ While the blood assay is the gold standard, non-invasive finger CO-oximetry has been touted as a potential cost-effective surrogate for screening.^16^–^18^ However, it is unclear how available either of these methods are to practicing clinicians. The goal of this study was to evaluate hospital capabilities of detecting carbon CO poisoning in three states in the upper Midwest.

## METHODS

We conducted a cross-sectional study of hospitals distributed over three Midwestern U.S. states (Minnesota, North Dakota, and South Dakota) served by both a single regional poison center – the Minnesota Poison Control System – and a single center for hyperbaric medicine with emergent treatment capabilities – the Hennepin County Medical Center Department of Undersea and Hyperbaric Medicine. We used multiple available sources, including state trauma databases, state health department websites, and the regional poison-center’s hospital database, to identify and compile all of the hospitals within the three-state area. All the identified hospitals were contacted by phone and surveyed from August 1, 2017 – May 3, 2018. Facilities were excluded if they did not have an emergency department (ED) (such as freestanding clinics) or if the hospital was no longer open.

We surveyed each facility in a standardized format regarding its ability to test for CO poisoning. Specific inquiries included whether the facility possessed in-house spectrophotometric HbCO assays, bedside CO-oximetry, or any manner to test for CO exposure on site. Additionally, facilities were queried regarding their use of send-out testing for CO exposure, as well as whether a process was in place to facilitate real-time testing.

**Population Health Research Capsule**

What do we already know about this issue?

*Carbon monoxide poisoning is one of the leading causes of poisoning in the United States*.

What was the research question?

*How available are methods for detecting carbon monoxide* (*CO) poisoning in hospitals in the upper Midwest?*

What was the major finding of the study?

*Hospitals serving smaller population areas are more likely to lack real-time testing for CO exposures*.

How does this improve population health?

*Understanding resource gaps could spur increased availability of point of care testing in smaller communities*.

We directed initial inquiries to the hospital-based clinical laboratory. A standardized greeting and introduction was followed by a simple query regarding capability to assay HbCO in the hospital lab, and a subsequent query with respect to the availability of bedside CO-oximetry at the facility. If the study inquiries were unanswered by laboratory staff or laboratory supervisor, a follow-up call to the ED was made. Following standardized introduction, a query was repeated with respect to the availability of bedside CO-oximetry to the supervising nurse on duty.

The reported populations of towns and cities housing each hospital were abstracted from the most recent United States Census Bureau dataset (USCB, 2010). These populations were divided into settlement hierarchy^19^ quartiles of ≤2,500, 2,501 – 25,000, 25,001 – 250,000, and ≥250,000 inhabitants with the assumption that hospitals in larger communities would be more likely to have a full range of care resources. Hospitals were further described with respect to their American College of Surgeons (ACS) trauma designation as an additional possible marker of available resources. For example, Level IV trauma center certification by the ACS requires 24-hour laboratory coverage, while Level V certification does not.^20^

Descriptive statistics characterizing study data were calculated in Stata/IC 15.0 for Mac (College Station, Texas). Relationships between the binary availability of HbCO testing and independent variables, including locale size, and American Trauma Society (ATS) trauma designation are reported using *x*^2^ or Fisher’s exact tests, as appropriate.

## RESULTS

**Figure 1:**
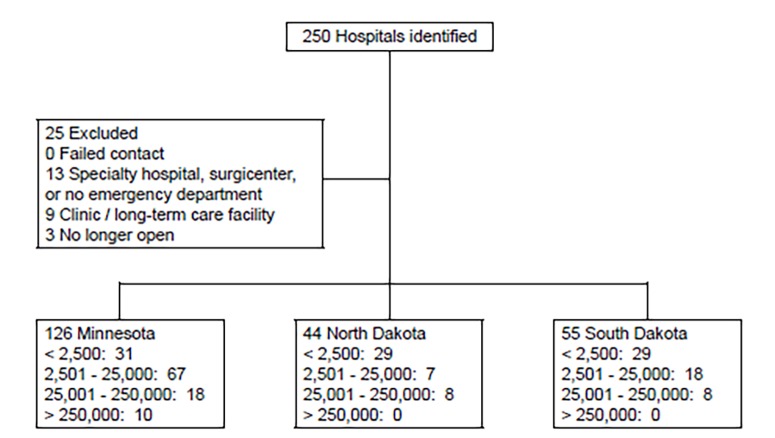
Study flow diagram of hospital capability to test for carbon monoxide poisoning.

We identified 250 facilities within the catchment area of the regional poison center and hyperbaric medicine unit. Included in the final analysis were 225 facilities (Table 1). Of the 25 facilities excluded, none were excluded due to failed contact, but three were excluded because the facility was no longer operational. Thirteen were specialty centers without a functioning ED, and nine were clinics or long-term care facilities (Figure 1). The population of the cities in which all hospitals were located, based on 2010 USCB results, ranged from 446 to 382,578 people.

**Figure 2:**
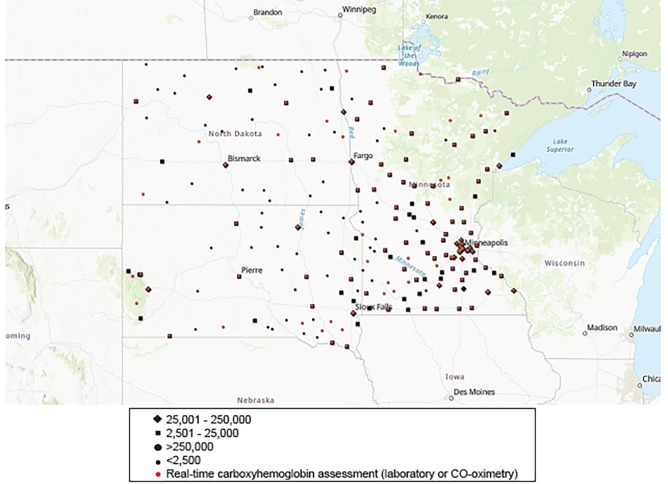
Distribution of responding hospitals and real-time carboxyhemoglobin monitoring by community size. *CO*, carbon monoxide.

Most facilities (181, 80.4%) were located in areas populated by less than 25,000 people. Hospital density per population was not equally distributed across the three states, with one for every 42,094 in Minnesota, one facility for every 15,286 inhabitants in North Dakota, and one for every 14,803 in South Dakota (Figure 2). Similarly, higher ATS trauma classification hospitals were more common to Minnesota than North Dakota or South Dakota.

Nearly all hospitals (n=220, 97.8%) reported some means of testing for CO poisoning (Table 2). A majority of facilities (n=133, 59.11%) reported some capacity for real-time testing. Facilities with more advanced trauma designations typically had greater ability to evaluate HbCO levels (Table 2). The proportion of hospitals capable of real-time HbCO measurement increased with population size from the lowest quartile at 32.6% to the highest quartile at 100% (Fisher’s exact test = 0.000). Smaller population size was associated with a higher proportion of hospitals reporting the use of send-out HbCO assays (Fisher’s exact test = 0.000). We also identified a strong association between reporting a lack of real-time testing and the use of send-out labs (Pearson’s *x*^2^ = 90, p = 0.000), an association that persisted across all hospital population strata.

## DISCUSSION

In this study of all hospitals in a three-state area, we found that most hospitals have some capacity for real-time testing of patients’ HbCO levels. However, smaller population areas were associated with gaps in real-time testing for HbCO and the use of send-out assays. Although it has been widely suspected that CO poisoning is underdiagnosed and under-reported in general, to our knowledge there have been no other studies looking at regional capabilities of detecting CO exposure and associated poisoning in the past decade, with only one similar study done in a different region of the U.S. in 2003 – 2004.^21^

A Centers for Disease Control and Prevention editorial noted concerns that CO poisoning may also be under-reported to poison centers in particular.^22^ Our data suggest that most hospitals in areas of less than 2500 people lack the ability to do real-time testing for CO exposure. Given that send-out assays often involve significant turnaround time and resources,^23^ it is possible that under-reporting and underdiagnosis of associated CO poisonings may be related to gaps in the capacity to detect HbCO levels.

The invisible nature of the gas and the vague presenting symptoms can make CO poisoning difficult to suspect and diagnose clinically, requiring a high degree of suspicion.^9^ Without a readily available means of testing, clinicians are unable to confirm the diagnosis. It is conceivable then that gaps in the regular availability of confirmatory testing might lead to cognitive biases^24^ that would prevent clinicians from suspecting or settling upon the diagnosis of CO poisoning in atypical presentations. Without suspicion or diagnosis, patients cannot be appropriately triaged or treated in a timely manner, whether with normobaric oxygen, hyperbaric oxygen, or other therapies. This study did not look into hospital referral patterns; however, previous studies have shown that 90% of patients referred for hyperbaric oxygen therapy come from facilities capable of testing in real time.^21^ Although referrals may be based on clinical suspicion, we suspect that this presents a clinical conundrum for both the referring clinician and the accepting facility.

Many of the facilities that we surveyed were in rural areas. Although facilities located in larger urban or suburban areas tended to possess better testing capabilities, rates of CO poisoning have been shown to be higher in rural areas.^4^ Work-related exposures and faulty furnaces account for significant sources of CO poisonings (45% in one study).^25^ Indeed, given current rural infrastructure and livelihoods, our concern is that individuals using gas heating implements or working on heavy and possibly running machinery in poorly-ventilated areas such as barns and sheds are more likely to be exposed, to go undiagnosed or be misdiagnosed, and to then return to the same practices that led to the exposure, compounding morbidity and increasing the likelihood of mortality from CO poisoning.

Historically, the majority of CO exposures in the U.S. have occurred in the Midwest, particularly accidental exposures.^22^ Indeed, sparse populations and rural areas with less infrastructure, particularly in North Dakota and South Dakota, do make these states distinct from much of the country. This area of the country also experiences significant cold-weather seasons, leading people to spend significant periods of time indoors with heaters, furnaces, and other sources of combustion, and it is during these colder months that the greatest number of poisonings occur.^4^–^6^ It is therefore of significant concern that many facilities in this upper Midwestern region do not have real-time capacity for detection of CO.

We believe that every hospital should possess some manner of real-time testing for CO poisoning. Delayed or missed diagnosis can have real effects on clinical outcomes.^11^ In addition, although prevention is key, all exposed patients should be afforded an opportunity to be appropriately evaluated for and diagnosed with CO poisoning so that they receive timely, appropriate treatment.

## LIMITATIONS

There are several limitations to this study. First, it is possible that we did not survey every hospital in the three states of concern. However, given our efforts to cross-reference multiple sources, we feel that this is a representative and nearly comprehensive sampling of hospitals in this geographic area. Second, it is possible that the individuals describing testing capabilities were inaccurate in their characterizations. However, we feel that the senior staff surveyed are likely to reflect a reasonable knowledge of the facility’s capabilities. Third, we did not quantify the turnaround time for send-out labs at each facility. Given that many of these hospitals are in remote areas, it is reasonable to assume that it would be at the very least several hours for results to return, especially when snowstorms and other weather events impact the region.

Additionally, we did not inquire about prehospital or out-of-hospital detection capacity or other established processes that might facilitate the diagnosis and treatment of CO poisoning, nor did we inquire about specific algorithms regarding the management of suspected CO poisoning, both of which are beyond the scope of this study. Finally, it is difficult to know if we can extrapolate the data from these three upper Midwestern states to the rest of the U.S. However, our data do compare favorably with a previous study.^21^ Additionally, if these gaps in testing capacity are present in areas with a high incidence of CO poisoning, they might well be suspected in areas of lower incidence across the country.

## CONCLUSION

In the geographic region encompassing Minnesota, North Dakota, and South Dakota, hospital-based and real-time capacity to test for CO exposure is not universal. In smaller population areas, hospitals are more likely to lack real-time testing or any testing at all. These findings may have significant public health, triage, and referral implications for patients who may be victims of CO exposure.

## Figures and Tables

**Table 1:** Distribution of responding hospitals.

City size	All states	Minnesota	North Dakota	South Dakota
Total n (%)*	225	126	44	55
<2,500	89 (39.6)	31 (24.6)	29 (65.9)	29 (52.7)
2,501 – 25,000	92 (40.9)	67 (53.2)	7 (15.9)	18 (32.7)
25,001 – 250,000	34 (15.1)	18 (14.3)	8 (18.2)	8 (14.6)
>250,000	10 (4.44)	10 (7.94)	0 (0.00)	0 (0.00)

* Percentage of responding hospitals located in cities of a given size.

**Table 2:** Availability of carboxyhemoglobin assessment.

	COHb lab assay	Finger CO-oximetry	Real-time COHb	Unable to test
Total n (%)	91 (29.6)	78 (25.4)	133 (43.3)	5 (1.62)
Population size				
<2,500	9 (10.1)	23 (25.8)	29 (32.6)	5 (5.62)
2,501 – 25,000	48 (52.2)	38 (41.3)	65 (70.7)	0 (0.00)
25,001 – 250,000	24 (70.6)	15 (44.1)	29 (85.3)	0 (0.00)
>250,000	10 (100.0)	2 (20.0)	10 (100.0)	0 (0.00)
ACS trauma designation				
I	5 (100.0)	4 (80.0)	5 (100.0)	0 (0.00)
II	12 (80.0)	5 (33.3)	15 (100.0)	0 (0.00)
III	25 (89.3)	12 (42.9)	27 (96.4)	0 (0.00)
IV	40 (35.4)	43 (38.1)	67 (59.3)	1 (0.88)
V	3 (7.0)	7 (16.3)	8 (18.6)	4 (9.30)
n/a	6 (28.6)	7 (33.3)	11 (54.2)	0 (0.00)

*COHb*, carboxyhemoglobin; *CO*, carbon monoxide; *ACS*, American College of Surgeons.

## References

[1] Raub JA, Mathieu-Nolf M, Hampson NB (2000). Carbon monoxide poisoning - a public health perspective. Toxicology.

[2] Hampson NB, Weaver LK (2007). Carbon monoxide poisoning: a new incidence for an old disease. Undersea Hyperb Med.

[3] Hampson NB (2016). U.S. mortality due to carbon monoxide poisoning, 1999–2014. Accidental and intentional deaths. Ann Am Thorac Soc.

[4] Mukhopadhyay S, Hirsch A, Etienne S (2018). Surveillance of carbon monoxide-related incidents — implications for prevention of related illnesses and injuries, 2005–2014. Am J Emerg Med.

[5] Stearns D, Sircar K (2018). National unintentional carbon monoxide poisoning estimates using hospitalization and emergency department data. Am J Emerg Med.

[6] (2017). QuickStats: Number of deaths resulting from unintentional carbon monoxide poisoning,* by month and year — National Vital Statistics System, United States, 2010–2015. MMWR Morb Mortal Wkly Rep.

[7] Weaver LK, Deru K, Churchill S, Legler J, Snow G, Grey T (2016). Carbon monoxide poisoning in Utah: 1996–2013. Undersea Hyperb Med.

[8] Hampson NB (2017). Cost effectiveness of residential carbon monoxide alarms. Undersea Hyperb Med.

[9] Ng PCY, Long B, Koyfman A (2018). Clinical chameleons: an emergency medicine focused review of carbon monoxide poisoning. Intern Emerg Med.

[10] Weaver LK (2009). Carbon monoxide poisoning. N Engl J Med.

[11] Weaver L, Hopkins R, Chan K (2002). Hyperbaric oxygen for acute carbon monoxide poisoning. N Engl J Med.

[12] Hampson NB, Piantadosi CA, Thom SR (2013). Practice recommendations in the diagnosis, management, and prevention of carbon monoxide poisoning. Diving Hyperb Med.

[13] CDC (2015). Clinical guidance for carbon monoxide (CO) poisoning after a disaster. Cdc.Gov.

[14] Johansson MB, Wollmer P (1989). Measurement of carboxyhaemoglobin by spectrophotometry and gas chromatography. Clin Physiol.

[15] Veronesi A, Pecoraro V, Zauli S (2017). Use of carboxyhemoglobin as a biomarker of environmental CO exposure: critical evaluation of the literature. Environ Sci Pollut Res.

[16] Zaouter C, Zavorsky GS (2012). The measurement of carboxyhemoglobin and methemoglobin using a non-invasive pulse CO-oximeter. Respir Physiol Neurobiol.

[17] Sebbane M, Claret P-G, Mercier G (2013). Emergency department management of suspected carbon monoxide poisoning: role of pulse CO-oximetry. Respir Care.

[18] Hullin T, Aboab J, Desseaux K (2017). Correlation between clinical severity and different non-invasive measurements of carbon monoxide concentration: a population study. PLoS One.

[19] Settlement characteristics - Revision 4- GCSE Geography - BBC.

[20] American Trauma Society (2016). Trauma center levels explained.

[21] Hampson NB, Scott KL, Zmaeff JL (2006). Carboxyhemoglobin measurement by hospitals: implications for the diagnosis of carbon monoxide poisoning. J Emerg Med.

[22] Centers for Disease Control and Prevention (CDC) (2011). Carbon Monoxide Exposures — United States, 2000–2009. MMWR Morb Mortal Wkly Rep.

[23] Clinical Laboratory and Standards Institute Selecting and Evaluating a Referral Laboratory 18 1998 11 1998. Available at: http://demo.nextlab.ir/getattachment/ac74de5f-6db3-47db-a5f3-37ec94625b63/CLSI-GP09-A.aspx Accessed January 24, 2019

[24] Croskerry P (2002). Achieving quality in clinical decision making: cognitive strategies and detection of bias. Acad Emerg Med.

[25] Grieb G, Simons D, Schmitz L (2011). Glasgow Coma Scale and laboratory markers are superior to COHb in predicting CO intoxication severity. Burns.

